# The cholesterol, high-density lipoprotein, and glucose index as a metabolic-nutritional biomarker for risk stratification in hospitalized heart failure patients

**DOI:** 10.3389/fnut.2026.1805016

**Published:** 2026-05-13

**Authors:** Zefan Huang, Liusheng Xu, Jinhui Zhang, Kefeng Lu, Jun Xu, Yichen Fang, Wei Ni, Ping Zhang, Tongbao Feng

**Affiliations:** 1 Institute of Reproductive Medicine, Medical School, Nantong University, Nantong, Jiangsu, China; 2 Department of Clinical Laboratory, The Second People's Hospital of Changzhou, The Third Affiliated Hospital of Nanjing Medical University, Changzhou, Jiangsu, China; 3 Department of Critical Care Medicine, The Affiliated Hospital of Jiangsu University, Zhenjiang, Jiangsu, China; 4 The First Clinical College, Xuzhou Medical University, Xuzhou, China

**Keywords:** CHG index, heart failure, hospital-based cohort, in-hospital mortality, metabolic-nutritional biomarker

## Abstract

**Background:**

Insulin resistance and dysregulated glucose-lipid metabolism contribute to adverse outcomes in heart failure (HF), yet existing metabolic indicators have limited utility for rapid risk stratification in critically ill patients. The cholesterol, high-density lipoprotein, and glucose (CHG) index may provide a convenient metabolic risk marker in hospitalized HF patients.

**Methods:**

This study adopted a dual-cohort retrospective design. The training cohort included 1,391 patients from the MIMIC-IV database (2008–2019), and the external validation cohort included 296 patients from an independent hospital-based cohort (July 2023 to December 2025). Patients were stratified into quartiles by CHG index. Kaplan–Meier survival analysis, Cox proportional hazards regression models, and restricted cubic spline (RCS) analysis were performed to assess the relationship between CHG index and in-hospital mortality.

**Results:**

In the training cohort, in-hospital mortality increased from 8.91% in Q1 to 17.87% in Q4 (log-rank test *p* = 0.0141). In the fully adjusted model, each 1-unit increase in CHG index was associated with a 76.8% increase in in-hospital mortality risk (HR = 1.768, 95% CI 1.337–2.338; *p* < 0.001). RCS analysis indicated an approximately linear positive association (*P* for overall < 0.001, *P* for nonlinearity = 0.139). The external validation cohort showed a similar association, with mortality increasing from 4.05% in Q1 to 16.22% in Q4 and a fully adjusted HR of 2.716 (95% CI 1.483–4.962, *p* = 0.001).

**Conclusion:**

The CHG index was independently associated with in-hospital mortality in patients with HF, with consistent findings in an external cohort. As an integrative biomarker derived from routine laboratory tests, the CHG index may reflect the interplay between metabolic dysregulation and cardiovascular vulnerability, thereby assisting early risk stratification in hospitalized patients with heart failure.

## 1 Introduction

Heart failure (HF) is a global public health challenge, affecting more than 64 million individuals worldwide and characterized by persistently elevated long-term mortality and rehospitalization rates (1, 2). Patients with HF admitted to the intensive care unit (ICU) experience particularly poor prognoses, with substantially higher in-hospital mortality rates, suggesting that metabolic derangements during hospitalization may contribute significantly to adverse clinical outcomes (3).

Insulin resistance (IR) serves as a critical metabolic hub in the pathogenesis and progression of HF. It is characterized by diminished systemic insulin sensitivity, which impairs glucose utilization and triggers compensatory hyperinsulinemia and hyperglycemia (4, 5). Through chronic inflammation and oxidative stress, IR exacerbates both lipotoxicity and glucotoxicity, driving cardiomyocyte hypertrophy, calcium dysregulation, and interstitial fibrosis. These pathological changes culminate in diastolic dysfunction and microcirculatory compromise, thereby substantially increasing the risk of adverse in-hospital outcomes. Concurrently, HF-associated neurohormonal activation together with hypoperfusion and hypoxia further aggravate IR, creating a vicious cycle that worsens prognosis (5–9). Emerging evidence suggests that metabolic dysregulation may also manifest through coronary microvascular dysfunction and altered cardiac electrophysiological stability, highlighting its broader cardiovascular relevance beyond conventional measures of insulin resistance (10, 11). Traditional IR assessment tools, including the homeostatic model assessment for insulin resistance (HOMA-IR), atherogenic index of plasma, triglyceride–glucose (TyG) index, and its derivative TyG-body mass index (TyG-BMI), exhibit notable limitations in HF risk stratification. These include dependence on insulin assays, inconsistent diagnostic thresholds across diverse populations and glycemic states, and vulnerability to confounding by comorbidities such as obesity, dyslipidemia, and renal impairment. Furthermore, the nonlinear or U-shaped associations reported in certain studies compromise their reliability and generalizability for HF risk assessment (5, 12–15). Consequently, there is an urgent need for a composite metabolic marker derived from routine laboratory tests that simultaneously captures glycemic burden and lipoprotein quality to enable rapid in-hospital risk evaluation and clinical decision-making (16, 17).

In this context, the cholesterol, high-density lipoprotein, and glucose (CHG) index, initially proposed by Mansoori and colleagues, is an emerging composite indicator that integrates total cholesterol (TC), high-density lipoprotein cholesterol (HDL-C), and fasting blood glucose (FBG) to provide a comprehensive assessment of metabolic homeostasis. In the setting of HF, it may also be viewed as an integrative indicator linking metabolic dysregulation with cardiovascular pathophysiology relevant to adverse clinical outcomes. Compared with conventional indices such as HOMA-IR, TyG index, and TyG-BMI, which primarily reflect insulin resistance or triglyceride–glucose interactions, the CHG index additionally incorporates HDL-C, a cardioprotective lipoprotein closely linked to endothelial homeostasis and metabolic health (18). This feature may allow the CHG index to capture not only glycemic and cholesterol-related burden, but also impairment in protective lipoprotein-related pathways relevant to HF risk stratification. Since its recent introduction, this index has demonstrated clinical utility in type 2 diabetes mellitus (T2DM), diabetic microvascular complications, hypertension, stroke, metabolic syndrome, and calcific aortic valve stenosis (CAVS), with favorable accessibility and cost-effectiveness (19–22). Given the central pathophysiological role of glucolipotoxicity in HF, the CHG index holds promise as an integrative risk indicator that may complement existing risk stratification approaches by reflecting not only metabolic burden, but also its potential cardiovascular implications in this population (4, 7, 23). However, to date, no study has systematically investigated the prognostic value of the CHG index in critically ill patients with HF, nor has its performance been validated across independent cohorts.

From a nutritional standpoint, the CHG index integrates three metabolically interrelated parameters that may be influenced by dietary factors: TC and HDL-C concentrations are modulated by the quality and quantity of dietary fat intake, whereas glucose homeostasis is regulated by carbohydrate consumption patterns, meal timing, and overall dietary composition (24, 25). Accumulating evidence indicates that adherence to Mediterranean dietary patterns, the Dietary Approaches to Stop Hypertension (DASH) regimen, and low-carbohydrate nutritional strategies can beneficially alter these metabolic parameters and attenuate cardiometabolic risk (25–27). Nevertheless, the potential utility of the CHG index as a nutritional biomarker to inform targeted dietary interventions in hospitalized HF patients remains unexplored.

Building on these considerations, we hypothesized that the CHG index, as an integrative biomarker, would be positively associated with in-hospital all-cause mortality in patients with HF and could reflect the convergence of metabolic dysregulation, cardiovascular vulnerability, and adverse clinical outcomes beyond conventional risk factors. Because the CHG index incorporates metabolic parameters related to glycemic and lipid status, demonstrating its prognostic value may support its potential role in risk stratification in HF management. To test this hypothesis, we conducted a dual-cohort retrospective study using data from the Medical Information Mart for Intensive Care IV (MIMIC-IV, version 3.1) database and an independent hospital-based validation cohort to evaluate the prognostic performance of the CHG index for in-hospital mortality risk stratification in patients with HF. Our findings may also provide a basis for future studies exploring the clinical relevance of the CHG index in metabolic and nutrition-related assessment among hospitalized HF patients.

## 2 Methods

### 2.1 Study design

This retrospective dual-cohort study was designed to evaluate and validate the association between the CHG index and in-hospital all-cause mortality among patients with HF, with a focus on assessing the index’s reproducibility and generalizability across distinct clinical settings. The study was conducted in accordance with the Strengthening the Reporting of Observational Studies in Epidemiology (STROBE) guidelines and the Declaration of Helsinki. The training cohort was derived from the MIMIC-IV database (version 3.1), a publicly accessible repository of de-identified critical care electronic health records. The database was developed through a collaboration between the Massachusetts Institute of Technology and Beth Israel Deaconess Medical Center (BIDMC) and contains clinical data for patients admitted to the ICU of the center from 2008 to 2019. All personal identifiers were removed through a rigorous de-identification process. In compliance with BIDMC Institutional Review Board regulations, the requirement for informed consent was waived, and data sharing for research purposes was authorized (28). Data extraction was performed by author Jinhui Zhang, who obtained the necessary access credentials (certificate number: 65008026).

The external validation cohort comprised single-center hospitalized patients identified through the Laboratory Information System (LIS) and Electronic Medical Record System (EMRS) of the Third Affiliated Hospital of Nanjing Medical University, spanning the period from July 2023 to December 2025. The study protocol received approval from the Clinical Research Ethics Committee of the hospital (approval number: [2025] KY115-01). Given the retrospective design and the use of de-identified data, the requirement for informed consent was waived.

### 2.2 Study participants and data extraction

Unified inclusion and exclusion criteria were applied to both cohorts. Hospitalized patients with HF were initially identified using diagnostic codes from the International Classification of Diseases, Ninth and Tenth Revisions (ICD-9 and ICD-10) (29). For patients with multiple hospitalizations, only the first admission was included in the analysis. Inclusion criteria required patients to be aged ≥ 18 years and have complete laboratory data for all three components of the CHG index (TC, HDL-C, and FBG), which were obtained within 24 h of hospital admission. Exclusion criteria comprised hospital length of stay < 24 h, concomitant acute stroke, active malignancy, or confirmed autoimmune disease.

**Figure 1:**
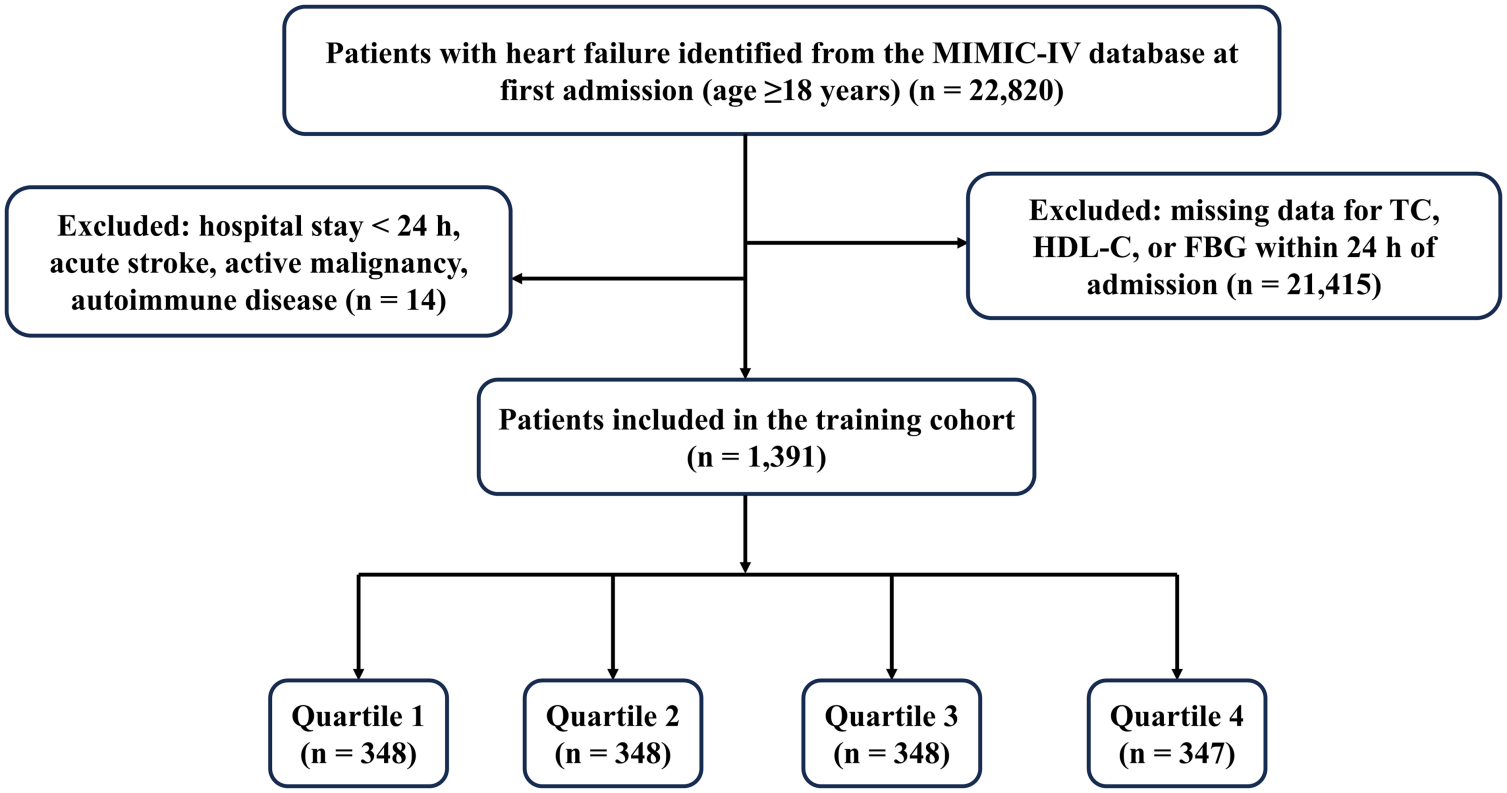
Flowchart of patient selection from the MIMIC-IV database.

In the MIMIC-IV database, 22,820 hospitalized patients with HF were initially screened. After applying exclusion criteria and removing cases with incomplete CHG index data within the first 24 h of admission, 1,391 patients were retained for analysis as the training cohort (patient selection flowchart shown in Figure 1). From the hospital database, 306 patients with HF were initially identified. Following application of identical exclusion criteria, 296 patients constituted the external validation cohort. To ensure consistency and comparability across the two cohorts, all laboratory variables used in the analyses, including TC, HDL-C, and FBG for CHG index calculation, were uniformly derived from the first measurements obtained within 24 h after hospital admission.

Variables were systematically extracted using standardized protocols in both cohorts and categorized into five domains: (1) Demographics: age, sex, race (with Han Chinese ethnicity recorded by default in the external validation cohort), and BMI; (2) Comorbidities: hypertension, diabetes, prior myocardial infarction, renal disease, and chronic pulmonary disease; (3) Vital signs and disease severity scores: heart rate, systolic blood pressure, diastolic blood pressure, oxygen saturation, Acute Physiology Score III (APS III), and Sequential Organ Failure Assessment (SOFA) score (APS III and SOFA score were not available in the external validation cohort due to differences in clinical documentation practices); (4) Laboratory tests: TC, triglyceride (TG), low-density lipoprotein cholesterol (LDL-C), HDL-C, glucose, glycated hemoglobin (HbA1c), troponin T (TNT), creatine kinase MB (CK-MB), albumin, serum creatinine (SCr), blood urea nitrogen (BUN), red blood cell count (RBC), hemoglobin (Hb), hematocrit, white blood cell count (WBC), and platelet (PLT); and (5) Clinical outcomes: in-hospital all-cause mortality and length of hospital stay.

The CHG index was calculated using the formula: CHG index = Ln [TC (mg/dL) × FBG (mg/dL) / (2 × HDL-C (mg/dL))] (19). To ensure unit consistency across cohorts, glucose and lipid measurements used in the analyses were standardized to mg/dL. In the external validation cohort, values originally reported in mmol/L were uniformly converted to mg/dL before CHG index calculation (conversion factors are provided in Supplementary Table S1). Patients were subsequently stratified into quartiles based on their CHG index on the first day of admission.

### 2.3 Outcomes

The primary endpoint was in-hospital all-cause mortality in both the training and validation cohorts.

### 2.4 Statistical analysis

For descriptive statistics, continuous variables were first assessed for normality using the Shapiro–Wilk test. Normally distributed data are presented as mean ± standard deviation and compared using Student’s t-test or analysis of variance (ANOVA). Non-normally distributed data are reported as median (interquartile range [IQR]) and compared using the Mann–Whitney *U* test. Categorical variables are expressed as frequencies (percentages) and compared using the χ^2^ test or Fisher’s exact test.

For survival analyses, participants were stratified into quartiles based on the CHG index. Kaplan–Meier survival curves were constructed, and between-group differences in cumulative survival probability were assessed using the log-rank test. Cox proportional hazards regression models were employed to evaluate the association between the CHG index and in-hospital mortality, with the index modeled both as a continuous variable (per 1-unit increment) and as a categorical variable (quartiles). Results are presented as hazard ratio (HR) with 95% confidence interval (CI), using the first quartile (Q1 group) as the reference category. Covariates were selected based on clinical relevance and univariable screening. Variables with *p* < 0.05 in univariable Cox regression analyses, along with clinically important variables regardless of statistical significance, were included in multivariable models. Univariable Cox regression analyses are presented in Table 1. Three progressively adjusted multivariable models were constructed: Model 1 included the CHG index alone (unadjusted); Model 2 adjusted for sex and age; Model 3 further adjusted for hypertension, diabetes, prior myocardial infarction, renal disease, chronic pulmonary disease, serum potassium, serum sodium, SCr, BUN, prothrombin time (PT), Hb, and PLT. Because the CHG index could only be calculated in patients with complete TC, HDL-C, and FBG measurements within 24 h of admission, the analyses were conducted using a complete-case approach.

**Table 1:** Univariable cox regression analyses for in-hospital mortality in training cohort.

Variables	HR	95% CI	*p* value
CHG index	1.596	1.265–2.014	<0.001
Male	0.877	0.653–1.177	0.381
Age	1.023	1.011–1.035	<0.001
Hypertension	0.731	0.497–1.077	0.113
Diabetes	1.383	1.033–1.851	0.030
Prior myocardial infarct	0.810	0.603–1.086	0.159
Renal disease	1.273	0.946–1.713	0.111
Chronic pulmonary disease	1.083	0.782–1.500	0.632
Potassium	1.329	1.122–1.574	<0.001
Sodium	1.018	0.987–1.050	0.260
SCr	1.107	1.017–1.205	0.018
BUN	1.013	1.008–1.018	<0.001
PT	1.021	1.010–1.032	<0.001
Hb	0.963	0.904–1.025	0.238
PLT	1.000	0.998–1.001	0.675

CHG index, cholesterol, high-density lipoprotein, and glucose index; HR, hazard ratio; CI, confidence interval; SCr, serum creatinine; BUN, blood urea nitrogen; PT, prothrombin time; Hb, hemoglobin; PLT, platelet.

Restricted cubic spline (RCS) analysis with four knots (at the 5th, 35th, 65th, and 95th percentiles) was performed to explore the potential dose–response relationship between the CHG index and in-hospital mortality. Subgroup analyses were performed to assess the robustness of the association between the CHG index and in-hospital mortality across clinically relevant strata, including sex (female or male), age (≤75 or >75 years), smoking status (yes or no), hypertension (yes or no), diabetes (yes or no), prior myocardial infarction (yes or no), and renal disease (yes or no). In each subgroup, the association was evaluated using Cox proportional hazards models with the CHG index entered as a continuous variable, and interaction tests were performed by including multiplicative interaction terms between the CHG index and each subgroup variable. *p* values for interaction were reported accordingly.

In the training cohort, to evaluate whether the CHG index provided additional predictive value beyond the TyG index, we compared the discriminatory and reclassification performance of models incorporating the CHG index with those incorporating the TyG index. Discrimination was assessed using the C-statistic, expressed as the area under the receiver operating characteristic curve (AUC), and reclassification was evaluated using the net reclassification improvement (NRI) and integrated discrimination improvement (IDI). In addition, to further assess the predictive performance and potential clinical usefulness of the CHG-based model, we examined its calibration and clinical utility in the training cohort. Calibration was assessed by comparing predicted and observed probabilities using a calibration plot, and clinical utility was assessed using decision curve analysis (DCA). In the DCA, model 1 represented the baseline model, and model 2 represented the baseline model plus the CHG index. All statistical analyses were performed using SPSS (version 26.0, IBM Corporation, USA) and R software (version 4.3.2, R Foundation for Statistical Computing, Vienna, Austria). A two-sided *p* value < 0.05 was considered statistically significant.

## 3 Results

### 3.1 Participant characteristics

Baseline characteristics of participants stratified by CHG index quartiles in the training cohort are presented in Supplementary Table S2. The training cohort comprised 1,391 hospitalized patients with HF from the MIMIC-IV database, stratified by quartiles of the CHG index measured within 24 h of admission (Q1: 3.24–5.08; Q2: 5.09–5.43; Q3: 5.44–5.84; Q4: 5.85–7.81). The median age was 73.23 years (IQR, 63.36–82.93), with 55.43% being male. Across ascending CHG index quartiles, notable demographic shifts were observed: age declined from 78.41 years in Q1 to 67.19 years in Q4 (*p* < 0.001), and male predominance increased from 48.28 to 62.25% (*p* < 0.001). The prevalence of obesity (BMI > 30 kg/m^2^) rose from 11.49 to 31.12% (*p* < 0.001).

**Figure 2:**
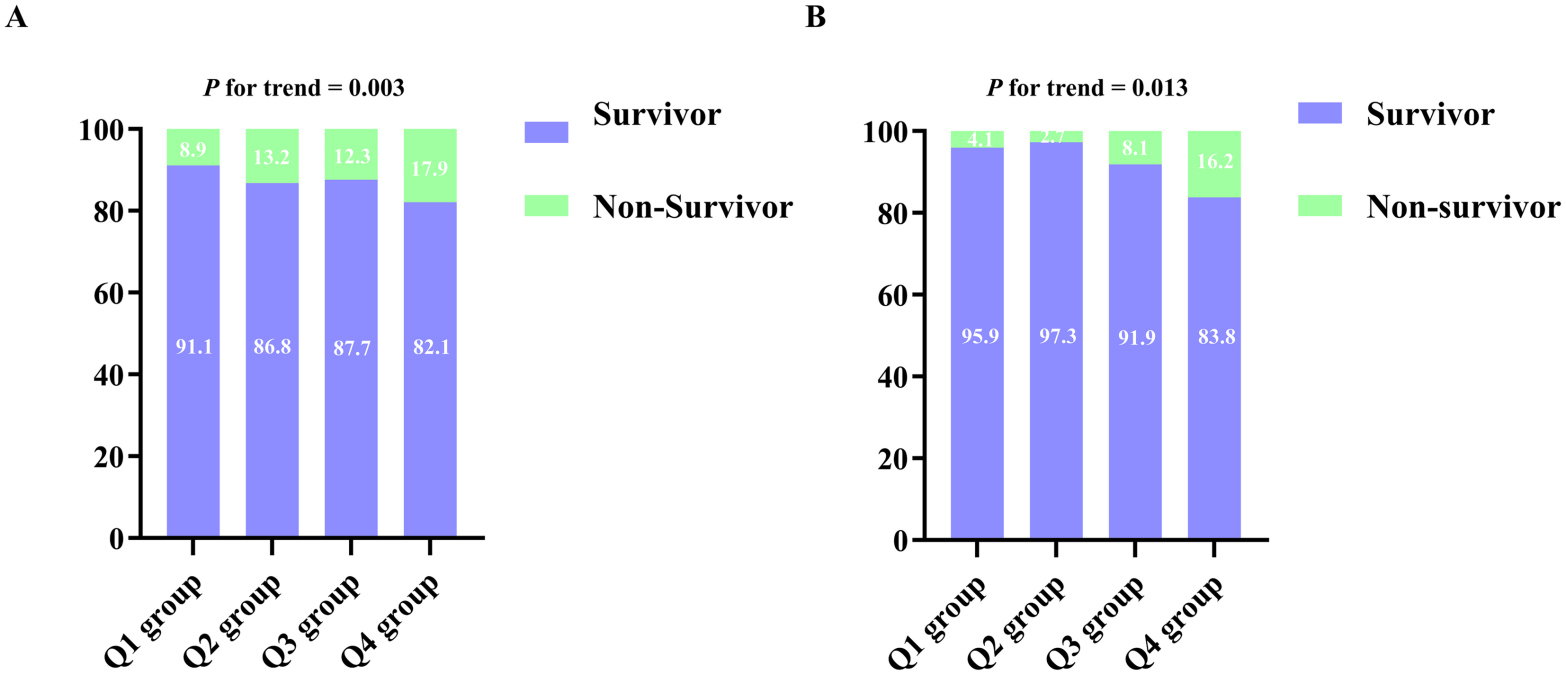
Proportion of in-hospital mortality across quartiles of the CHG index in both cohorts. **(A)** Training cohort (MIMIC-IV, *n* = 1,391), CHG index quartiles: Q1 (3.24–5.08), Q2 (5.09–5.43), Q3 (5.44–5.84), Q4 (5.85–7.81); **(B)** Validation cohort (hospital-based, *n* = 296), CHG index quartiles: Q1 (3.82–5.24), Q2 (5.25–5.51), Q3 (5.52–5.94), Q4 (5.95–6.85).

With respect to vital signs and disease severity, heart rate increased progressively across quartiles (*p* < 0.001), as did APS III scores (*p* < 0.001), whereas SOFA scores showed variability without a clear linear trend (*p* = 0.009). Laboratory parameters reflected escalating glucose–lipid dysregulation and heightened inflammatory–injury burden in higher CHG index groups: TC, TG, LDL-C, glucose, and HbA1c all increased, while HDL-C decreased (all *p* < 0.001); additionally, TNT, CK-MB, WBC, PLT, RBC, Hb, and hematocrit were progressively elevated (all *p* < 0.001). Regarding renal function, SCr showed modest incremental increases across quartiles (*p* < 0.001), whereas BUN showed no significant differences. In-hospital mortality increased from 8.91% in the Q1 group to 17.87% in the Q4 group (*p* = 0.006, Figure 2A).

Baseline characteristics of the external validation cohort are summarized in Supplementary Table S3. This cohort included 296 HF inpatients from our institution, stratified by CHG index quartiles (Q1: 3.82–5.24; Q2: 5.25–5.51; Q3: 5.52–5.94; Q4: 5.95–6.85). The median age (75.50 years) was marginally higher than that in the training cohort but similarly decreased with ascending CHG index quartiles (*p* < 0.05). Metabolic profiles mirrored those observed in the training cohort: TC, TG, LDL-C, glucose, and HbA1c increased across quartiles, concomitant with declining HDL-C (all *p* < 0.001). With regard to inflammatory and hemostatic markers, both PLT and WBC demonstrated progressive elevation with higher quartiles (*p* < 0.001 and *p* = 0.006, respectively). SCr increased incrementally across quartiles (*p* < 0.05), whereas BUN and albumin showed no significant inter-group differences. In-hospital mortality escalated from 4.05% in Q1 to 16.22% in Q4 (*p* = 0.010, Figure 2B).

### 3.2 Primary outcome

**Figure 3:**
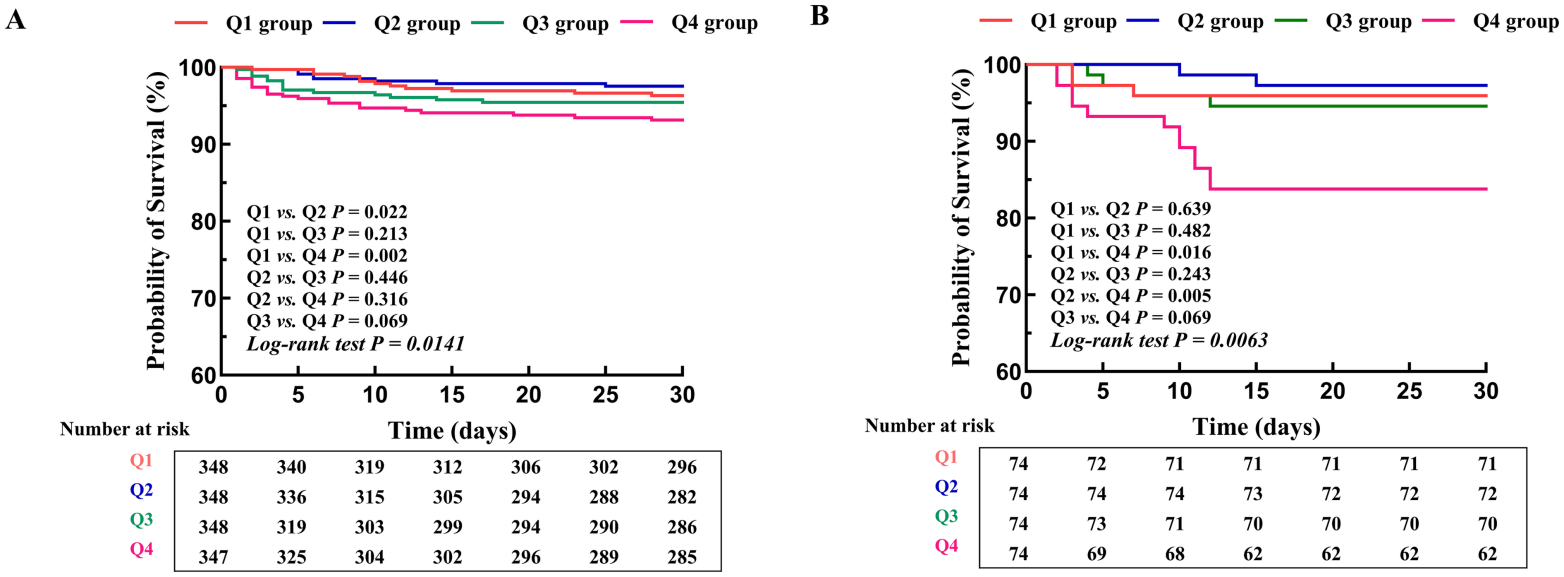
Kaplan–Meier survival curves stratified by CHG index quartiles in both cohorts. **(A)** Training cohort (MIMIC-IV, *n* = 1,391), CHG index quartiles: Q1 (3.24–5.08), Q2 (5.09–5.43), Q3 (5.44–5.84), Q4 (5.85–7.81), log-rank test *p* = 0.0141; **(B)** Validation cohort (hospital-based, *n* = 296), CHG index quartiles: Q1 (3.82–5.24), Q2 (5.25–5.51), Q3 (5.52–5.94), Q4 (5.95–6.85), log-rank test *p* = 0.0063.

Kaplan–Meier survival curves stratified by CHG index quartiles are presented in Figure 3. In the training cohort, survival probabilities differed significantly across quartiles (log-rank test *p* = 0.0141, Figure 3A). The Q4 group exhibited the steepest decline in survival probability, with pronounced early drops that persisted throughout hospitalization, followed by Q3. In contrast, Q1 and Q2 groups maintained consistently higher survival probabilities with more gradual declines. The validation cohort demonstrated a comparable pattern (log-rank test *p* = 0.0063, Figure 3B), with Q4 showing the lowest survival probability and the most rapid early decline.

Cox proportional hazards regression analyses demonstrated a significant association between the CHG index and in-hospital mortality. In the training cohort (Table 2), after full adjustment, each 1-unit increment in CHG index conferred a 76.8% elevated mortality risk (HR = 1.768, 95% CI 1.337–2.338; *p* < 0.001). When analyzed by quartiles, a significant dose–response relationship was observed (*P* for trend = 0.003), with patients in the highest quartile (Q4) showing a significantly elevated risk (HR = 2.413, 95% CI 1.451–4.011; *p* < 0.001) compared with Q1. These findings were corroborated in the validation cohort (Table 3), where each 1-unit increase in CHG index was associated with a fully adjusted HR of 2.716 (95% CI 1.483–4.962; *p* = 0.001). The validation cohort also demonstrated a consistent dose–response relationship (*P* for trend = 0.001), with Q4 showing a significant association compared with Q1 (HR = 15.391, 95% CI 2.752–86.025; *p* = 0.002).

**Table 2:** Cox regression analysis of CHG index and in-hospital mortality in training cohort.

Variables	Model1		*P* for trend	Model2		*P* for trend	Model3		*P* for trend
	HR (95% CI)	*p* value		HR (95% CI)	*p* value		HR (95% CI)	*p* value	
Hospital mortality									
Continuous variable per unit	1.596 (1.265–2.014)	<0.001		1.901 (1.487–2.430)	<0.001		1.768 (1.337–2.338)	<0.001	
Quartile			0.007			<0.001			0.003
Q1 group	1.000 (Reference)			1.000 (Reference)			1.000 (Reference)		
Q2 group	1.611 (1.021–2.541)	0.040		1.670 (1.058–2.635)	0.028		1.858 (1.152–2.997)	0.011	
Q3 group	1.350 (0.850–2.146)	0.204		1.482 (0.930–2.359)	0.098		1.444 (0.878–2.375)	0.148	
Q4 group	1.929 (1.251–2.973)	0.003		2.454 (1.572–3.833)	<0.001		2.413 (1.451–4.011)	<0.001	

Model1: Unadjusted model.

Model2: Adjusted for sex and age.

Model3: Adjusted for sex, age, hypertension, diabetes, prior myocardial infarct, renal disease, chronic pulmonary disease, potassium, sodium, SCr, BUN, PT, Hb, PLT.

CHG index, cholesterol, high-density lipoprotein, and glucose index; HR, hazard ratio; CI, confidence interval; SCr, serum creatinine; BUN, blood urea nitrogen; PT, prothrombin time; Hb, hemoglobin; PLT, platelet.

**Table 3:** Cox regression analysis of CHG index and in-hospital mortality in validation cohort.

Variables	Model1		*P* for trend	Model2		*P* for trend	Model3		*P* for trend
	HR (95%CI)	*p* value		HR (95%CI)	*p* value		HR (95%CI)	*p* value	
Hospital mortality									
Continuous variable per unit	1.793 (1.181–2.724)	0.006		2.083 (1.362–3.181)	<0.001		2.716 (1.483–4.962)	0.001	
Quartile			0.006			<0.001			0.001
Q1 group	1.00 (Reference)			1.00 (Reference)			1.00 (Reference)		
Q2 group	0.723 (0.124–4.336)	0.716		1.181 (0.182–7.813)	0.864		0.763 (0.102–5.713)	0.792	
Q3 group	2.092 (0.532–8.394)	0.298		3.823 (0.841–17.274)	0.082		3.241 (0.701–15.123)	0.134	
Q4 group	4.063 (1.133–14.501)	0.031		7.331 (1.803–29.903)	0.005		15.391 (2.752–86.025)	0.002	

Model1: Unadjusted model.

Model2: Adjusted for sex and age.

Model3: Adjusted for sex, age, hypertension, diabetes, prior myocardial infarct, renal disease, chronic pulmonary disease, potassium, sodium, SCr, BUN, PT, Hb, PLT.

CHG index, cholesterol, high-density lipoprotein, and glucose index; HR, hazard ratio; CI, confidence interval; SCr, serum creatinine; BUN, blood urea nitrogen; PT, prothrombin time; Hb, hemoglobin; PLT, platelet.

**Figure 4:**
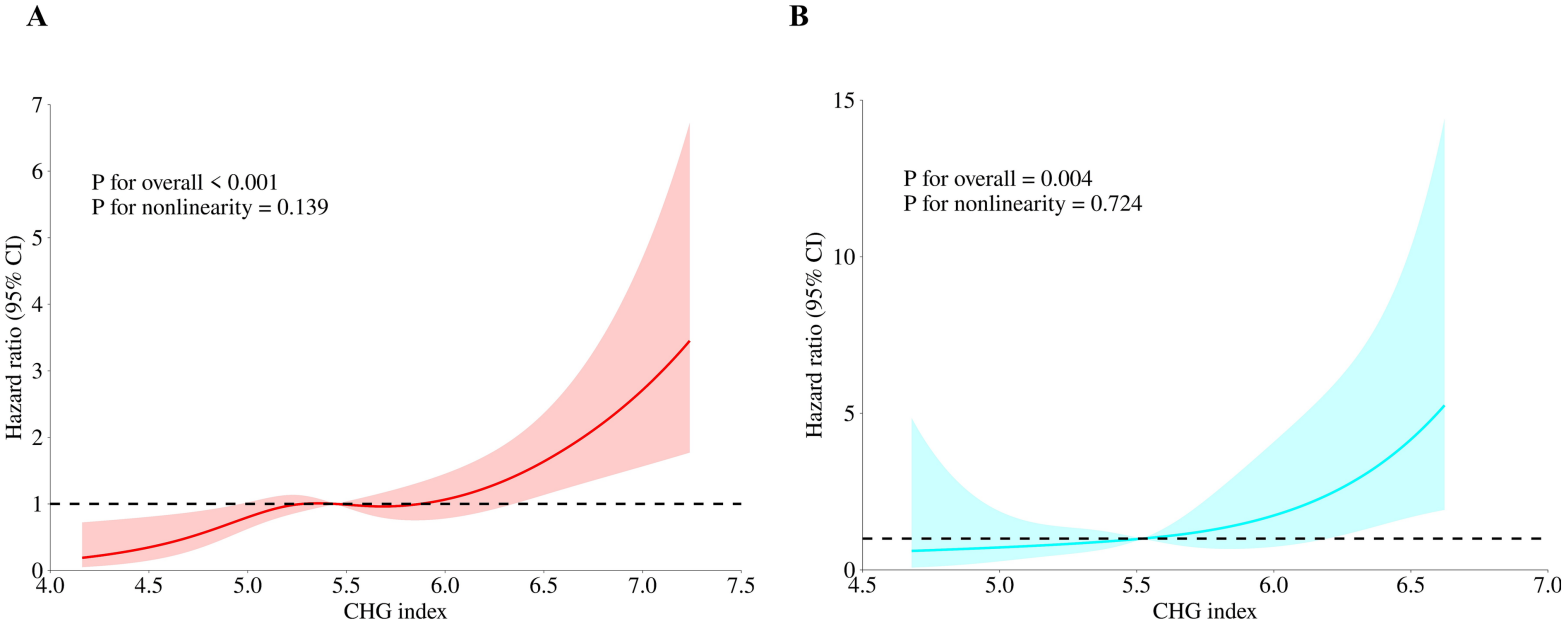
Dose–response relationship between CHG index and in-hospital mortality in both cohorts. Solid line: hazard ratio; shaded area: 95% CI; horizontal dotted line: hazard ratio of 1.0. **(A)** Training cohort (MIMIC-IV, *n* = 1,391), *P* for overall < 0.001, *P* for nonlinearity = 0.139; **(B)** Validation cohort (hospital-based, *n* = 296), *P* for overall = 0.004, *P* for nonlinearity = 0.724. CHG index, cholesterol, high-density lipoprotein, and glucose index; CI, confidence interval.

RCS analysis examining the dose–response relationship between CHG index and in-hospital mortality is depicted in Figure 4. In the training cohort, RCS revealed a significant positive association (*P* for overall < 0.001, Figure 4A), with no evidence of nonlinearity (*P* for nonlinearity = 0.139), indicating an approximately linear dose–response relationship without an identifiable threshold. The validation cohort corroborated these findings, demonstrating a consistent positive association with no detectable nonlinear pattern (*P* for overall = 0.004, *P* for nonlinearity = 0.724, Figure 4B), further supporting the linear dose–response relationship.

**Figure 5:**
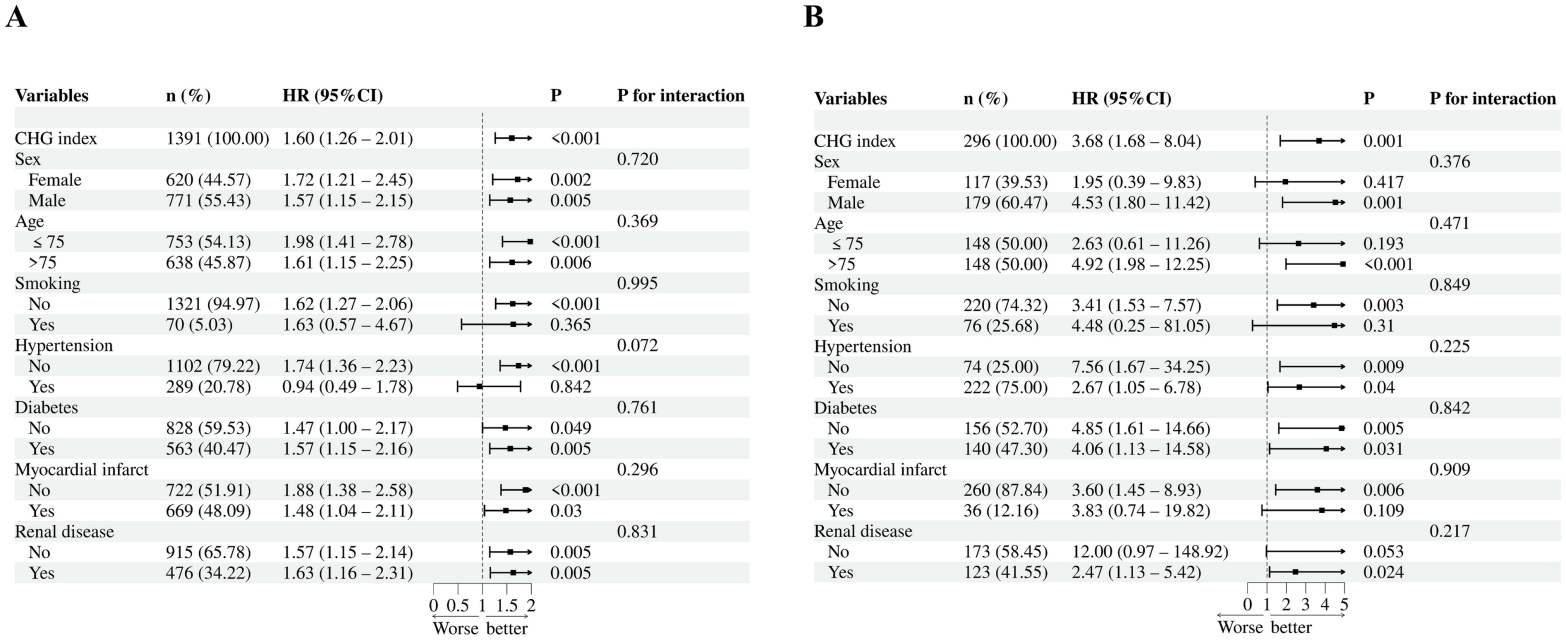
Subgroup analyses of CHG index and in-hospital mortality in both cohorts. **(A)** Training cohort (MIMIC-IV, *n* = 1,391), all *P* for interaction > 0.05; **(B)** Validation cohort (hospital-based, *n* = 296), all *P* for interaction > 0.05. Solid square: point estimates of HRs; horizontal line: 95% CI; vertical dashed line: hazard ratio of 1.0. *p* values for each subgroup and interaction tests are displayed on the right. CHG index, cholesterol, high-density lipoprotein, and glucose index; HR, hazard ratio; CI, confidence interval.

Subgroup analyses examining the association between the CHG index and in-hospital mortality are presented in Figure 5. In the training cohort, the direction of association remained generally consistent across subgroups defined by sex, age, smoking status, hypertension, diabetes, prior myocardial infarction, and renal disease, and no significant interactions were observed (all *P* for interaction > 0.05, Figure 5A). Similar findings were observed in the validation cohort (all *P* for interaction > 0.05, Figure 5B).

### 3.3 Incremental predictive value and model performance of the CHG index

To further evaluate the incremental predictive value of the CHG index for risk prediction, we compared the discriminative and reclassification performance of the baseline model with that of extended models incorporating either the TyG index or the CHG index. As shown in Supplementary Table S4, the baseline model yielded an AUC of 0.683 (95% CI 0.641–0.725). After addition of the TyG index, the AUC increased to 0.697 (95% CI 0.657–0.738), corresponding to a ΔAUC of 0.014. After addition of the CHG index, the AUC increased to 0.701 (95% CI 0.659–0.742), corresponding to a ΔAUC of 0.018. Reclassification analyses are presented in Supplementary Table S5. Compared with the baseline model, the model including the TyG index yielded a categorical NRI of 0.018 (95% CI −0.013–0.049; *p* = 0.256), a continuous NRI of 0.228 (95% CI 0.068–0.389; *p* = 0.005), and an IDI of 0.010 (95% CI 0.004–0.017; *p* = 0.003). The model including the CHG index showed a categorical NRI of 0.033 (95% CI −0.005–0.072; *p* = 0.091), a continuous NRI of 0.171 (95% CI 0.011–0.331; *p* = 0.036), and an IDI of 0.019 (95% CI 0.010–0.029; *p* < 0.001). Overall, the CHG index showed marginally better incremental predictive performance than the TyG index, as reflected by a slightly greater increase in AUC and a higher IDI.

The performance of the CHG-based model is further presented in Supplementary Figure S1. The receiver operating characteristic curve showed a C-statistic of 0.701 (95% CI 0.659–0.742) (Supplementary Figure S1A). DCA indicated that the CHG-based model (model 2) provided a modest net clinical benefit compared with the baseline model (model 1), particularly at lower and intermediate threshold probabilities (Supplementary Figure S1B). The calibration plot showed acceptable agreement between predicted and observed probabilities (Supplementary Figure S1C).

## 4 Discussion

This dual-cohort study represents the first systematic evaluation of the CHG index as a prognostic biomarker in hospitalized HF patients. Across both the MIMIC-IV training cohort and the external validation cohort, the CHG index demonstrated a consistent, approximately linear association with in-hospital all-cause mortality, as evidenced by Cox regression analyses, Kaplan–Meier survival analyses, and RCS analyses. Subgroup analyses further confirmed the robustness of this association across diverse clinical subsets.

In addition, the CHG index showed marginally better incremental predictive performance than the TyG index, with a slightly greater increase in AUC and a higher IDI. The CHG-based model also demonstrated a C-statistic of 0.701, acceptable calibration, and a modest net clinical benefit on DCA, particularly at lower and intermediate threshold probabilities. Together, these findings suggest that the CHG index may provide added prognostic information beyond conventional clinical variables in hospitalized HF patients. Given that the CHG index is derived from routine metabolic parameters that may be influenced by nutritional and metabolic status, it may also have potential clinical relevance for individualized risk evaluation in this population.

IR and its associated metabolic phenotypes represent key pathophysiological determinants of HF progression and adverse outcomes (5, 12). Prior investigations have demonstrated that the TyG index and related indices, serving as surrogate measures of IR, possess prognostic utility in cardiovascular risk stratification. However, these metrics predominantly capture glycemic-lipid metabolic burden while inadequately reflecting lipoprotein functionality and reverse cholesterol transport capacity (13, 30–34). Similarly, although the stress hyperglycemia ratio independently predicts 28-day mortality in ICU patients with pre-existing chronic HF, its exclusive focus on acute glycemic perturbations and dependence on HbA1c measurement constrain its clinical applicability (3). Beyond their prognostic limitations, these indices offer little guidance for nutritional management. They fail to distinguish between modifiable lipid quality (e.g., HDL-C) and overall lipid burden, nor do they enable targeted dietary strategies aimed at correcting specific metabolic derangements. These shortcomings underscore the critical need for a composite metabolic marker that simultaneously integrates glycemic burden and lipoprotein quality while maintaining accessibility for routine clinical implementation.

Compared with traditional IR-related indices, the CHG index may provide additional metabolic information by incorporating HDL-C. Mansoori et al. initially proposed this index as a screening tool for T2DM, demonstrating superior discriminative capacity relative to the TyG index. Subsequent studies have further expanded the clinical utility of the CHG index, validating its independent predictive value not only for diabetic nephropathy and retinopathy in patients with established T2DM but also for the risk of hypertension, stroke, metabolic syndrome, and CAVS (19–22). Emerging evidence now suggests broader cardiovascular applications. Recently, Mo et al. reported that each 1-unit increase in the CHG index conferred a 20% increased risk of cardiovascular events in the China Health and Retirement Longitudinal Study cohort (HR = 1.20, 95% CI 1.07–1.34), demonstrating marginally superior predictive performance compared with the TyG index (HR = 1.08, 95% CI 1.01–1.17) (23). Nevertheless, its prognostic value for short-term mortality in hospitalized HF patients has not been well characterized. In this study, the CHG index was independently associated with short-term mortality across two cohorts, extending current evidence to this high-risk clinical setting. From this perspective, the CHG index may be better understood not merely as a metabolic marker, but as an integrative indicator related to metabolic dysregulation, cardiovascular vulnerability, and adverse clinical outcomes in hospitalized patients with HF.

Notably, we observed an approximately linear dose–response relationship between the CHG index and adverse clinical outcomes, whereas the TyG index and TyG-BMI may exhibit nonlinearity, threshold effects, or paradoxical associations in HF populations (12, 13, 34–36). This distinction likely reflects the greater weighting of HDL-C within the CHG index, which more effectively captures declining vascular protection and impaired reverse cholesterol transport capacity, thereby yielding more stable prognostic performance during HF hospitalization (18). From a clinical nutrition standpoint, the weighting of HDL-C within the CHG index merits attention, as HDL functionality has been associated with Mediterranean dietary patterns, polyphenol-rich food intake, and optimized dietary fat composition, factors that may also be relevant to CHG index variation and clinical outcomes (25, 37). Furthermore, in the context of the “cholesterol paradox” observed in chronic or advanced HF—wherein lower TC or LDL-C paradoxically associates with increased mortality due to inflammatory catabolism or cardiac cachexia—the CHG index’s incorporation of HDL-C quality enables stable identification of high-risk individuals through integrated assessment of reduced high-density lipoprotein (HDL) functionality and hyperglycemia, thereby circumventing the potentially misleading interpretations arising from isolated reliance on cholesterol concentrations (17, 38).

Kaplan–Meier analyses showed significant differences in survival across CHG index quartiles (training cohort: log-rank test *p* = 0.0141; validation cohort: log-rank test *p* = 0.0063), and the curves separated early during hospitalization. The highest CHG quartile showed the lowest survival probability, suggesting that patients with a higher CHG index may warrant closer clinical attention early after admission. This pattern supports a potential role for the CHG index in early risk stratification and clinical evaluation during hospitalization.

Cox regression and RCS analyses showed a positive linear association between the CHG index and in-hospital mortality, with no clear evidence of a threshold or saturation effect. In multivariable Cox models adjusted for demographic characteristics, comorbidities, and baseline laboratory parameters, each 1-unit increase in the CHG index was associated with higher in-hospital mortality in both the training cohort (HR = 1.768, 95% CI 1.337–2.338) and the external validation cohort (HR = 2.716, 95% CI 1.483–4.962). Quartile-based analyses showed a similar pattern, with higher mortality risk across increasing CHG index quartiles in both cohorts. In the training cohort, patients in the highest quartile had a significantly higher mortality risk than those in the lowest quartile (HR = 2.413, 95% CI 1.451–4.011; *P* for trend = 0.003). A comparable monotonic gradient was observed in the validation cohort (Q4 vs. Q1: HR = 15.391, 95% CI 2.752–86.025; *P* for trend = 0.001). These findings suggest that the CHG index may have prognostic value in different cohorts, although the effect size varied between cohorts. However, these external validation findings should be interpreted cautiously. The validation cohort was relatively small and derived from a single center, and important severity scores such as APS III and SOFA score were unavailable, which may have limited risk adjustment and reduced the strength of inference regarding broader generalizability. The larger effect size observed in the validation cohort may reflect the smaller sample size and lower event number, which can lead to less stable estimates and wider CIs, as well as differences in baseline patient characteristics, laboratory measurement platforms, and in-hospital therapeutic pathways between the two cohorts. Therefore, the external validation in this study should be considered supportive rather than definitive, and future multicenter prospective investigations with standardized laboratory protocols and more comprehensive severity assessment are needed to further confirm the generalizability of these findings.

Having established the prognostic value of the CHG index, we now consider potential mechanistic underpinnings. Throughout the development and progression of HF, metabolic reprogramming, excessive neurohumoral activation, systemic chronic inflammation, and heightened oxidative stress have been extensively documented. These interconnected processes likely synergize to promote lipotoxic and glucotoxic myocardial injury (39–44). Under conditions of elevated metabolic burden reflected by increased CHG index, adverse outcomes may be mediated through several pathways. First, cholesterol accumulation and HDL dysfunction: Elevations in TC and LDL-C may intensify atherosclerotic burden and impair microvascular perfusion, whereas reductions in HDL-C may attenuate reverse cholesterol transport alongside diminished anti-inflammatory and antioxidant protective mechanisms (45–47). Second, the hyperglycemia–oxidative stress–mitochondrial axis: Stress hyperglycemia and IR may precipitate endothelial dysfunction, immunometabolic dysregulation, and disrupted calcium homeostasis, consequently triggering mitochondrial injury within cardiomyocytes (4, 5, 48, 49). Third, activation of the inflammation–coagulation cascade: Across both training and validation cohorts, we observed progressive increments in peripheral WBC and PLT counts with ascending CHG index quartiles, a pattern that may be consistent with greater systemic inflammation and coagulation activation among patients with higher metabolic burden. Excessive release of inflammatory mediators coupled with PLT activation may exacerbate endothelial injury, microvascular thrombosis, and pathological myocardial remodeling, thereby perpetuating disease progression (50–52). Importantly, each of these pathogenic pathways may be influenced by nutritional factors. Dietary patterns targeting lipid quality (e.g., replacing saturated with unsaturated fats) have been associated with more favorable lipid metabolism and HDL functionality. Glycemic optimization through dietary modification may help attenuate hyperglycemia-related oxidative stress. Anti-inflammatory dietary patterns, such as the Mediterranean diet rich in omega-3 fatty acids and polyphenols, have also been linked to lower systemic inflammation and coagulation activity. The CHG index may therefore help identify high-risk patients while highlighting metabolic pathways that could be relevant to future nutrition-related research (25, 53, 54).

Subgroup analyses across clinically relevant strata, including sex, age, smoking status, and major comorbidities (hypertension, diabetes, prior myocardial infarction, and renal disease), showed similar associations between the CHG index and in-hospital mortality in both cohorts (all *P* for interaction > 0.05; Figure 5). Notably, the association remained stable regardless of diabetes status (*P* for interaction = 0.761 and 0.842 in the training and validation cohorts, respectively) or renal disease (*P* for interaction = 0.831 and 0.217 in the training and validation cohorts, respectively), suggesting that the CHG index may reflect mortality risk across different clinical subgroups rather than only specific comorbidity groups. This may differ from traditional glycemic markers such as FBG or HbA1c, which are often considered more informative in patients with diabetes. The stability of associations across renal function strata merits particular emphasis, given that kidney impairment frequently perturbs both lipoprotein metabolism and glucose homeostasis. The consistency of the association across subgroups suggests that the prognostic value of the CHG index may be relatively stable across different clinical profiles.

A potential clinical relevance of the CHG index lies in its ability to reflect metabolic characteristics captured by routine laboratory parameters. All three components—TC, HDL-C, and FBG—reflect metabolic domains that are known to be influenced by dietary patterns and nutrient composition, including dietary fat quality and carbohydrate quality (25, 55). Rather than indicating direct treatment responsiveness, the CHG index may reflect a metabolic phenotype that is relevant to risk stratification. This composite design suggests that the CHG index may serve as a prognostic marker for identifying high-risk patients who may warrant closer metabolic and nutrition-related evaluation. Given that its components reflect glycemic and lipid-related metabolic status, the CHG index may have potential relevance to nutrition-oriented risk assessment. However, because this study did not include data on dietary intake, nutritional interventions, or longitudinal changes in the CHG index, the present findings do not support using the CHG index as a guide for specific nutritional interventions or therapeutic decision-making. Its clinical utility in nutrition practice therefore remains to be established in future prospective studies.

Several limitations should be noted. First, the retrospective design limits causal inference, and residual confounding may persist despite multivariable adjustment. In particular, several potentially important confounders, including in-hospital medication use (e.g., statins, sodium-glucose cotransporter 2 inhibitors, and insulin), baseline nutritional status (including malnutrition or cachexia), and ICU interventions, were not fully available or consistently recorded in the database and therefore could not be comprehensively adjusted for in the analyses. In addition, because calculation of the CHG index required complete TC, HDL-C, and FBG measurements within 24 h of admission, a substantial proportion of initially screened patients were excluded due to missing data. This complete-case approach may have introduced selection bias and may limit the representativeness of the final study population. These unmeasured factors may be associated with both CHG index levels and in-hospital mortality and may have influenced the observed association. Therefore, the CHG index may partly reflect overall disease severity rather than a fully independent prognostic factor. Accordingly, the findings should be interpreted cautiously as hypothesis-generating. Second, the CHG index was calculated using the first laboratory measurements obtained within 24 h of admission and thus reflects only an early single time-point assessment. Accordingly, it does not capture temporal metabolic fluctuations or dynamic changes in response to treatment during hospitalization. Future studies incorporating serial CHG measurements may help clarify whether longitudinal changes in the CHG index provide additional prognostic value or better reflect treatment response in hospitalized HF patients. Third, the outcome assessment in this study was limited to in-hospital all-cause mortality. Cause-specific outcomes were not available; therefore, we could not distinguish cardiac from non-cardiac deaths or perform cause-specific analyses. In addition, lack of echocardiographic data precluded phenotype-specific analyses (HFrEF, HFmrEF, and HFpEF), and incomplete availability of some clinical variables also precluded comparison with established HF risk scores. Fourth, although an external validation cohort was included, it was relatively small and derived from a single center, which may limit the precision and broader generalizability of the validation findings. In addition, important severity scores such as APS III and SOFA score were unavailable in the validation cohort, precluding fully comparable adjustment for illness severity between the two cohorts. Fifth, dietary intake data and nutritional interventions during hospitalization were not systematically recorded, precluding assessment of whether changes in CHG index resulted from dietary modifications or medical therapies. Future prospective studies should incorporate detailed nutritional assessment and track dietary adherence to better elucidate nutrition’s role in modulating CHG index trajectories and clinical outcomes.

## 5 Conclusion

Derived from routine laboratory tests, the CHG index was independently associated with in-hospital all-cause mortality and may be useful for early risk stratification in hospitalized HF patients. In this clinical setting, it may be understood as an integrative biomarker reflecting the relationship between metabolic dysregulation, cardiovascular vulnerability, and adverse clinical outcomes. Further prospective studies in diverse HF populations are needed to clarify its value in longitudinal evaluation and to validate its role as a metabolic-nutritional biomarker for risk stratification.

## Data Availability

The MIMIC-IV database (version 3.1) is publicly available. Requests to access these de-identified datasets should be directed to Liusheng Xu, liushengxu1024@163.com.

## References

[1] Savarese G Becher PM Lund LH Seferovic P Rosano GMC Coats AJS. Global burden of heart failure: a comprehensive and updated review of epidemiology. Cardiovasc Res. (2023) 118:3272–87. doi: 10.1093/cvr/cvac013, 35150240

[2] McDonagh TA Metra M Adamo M Gardner RS Baumbach A Bohm M . 2023 focused update of the 2021 esc guidelines for the diagnosis and treatment of acute and chronic heart failure. Eur Heart J. (2023) 44:3627–39. doi: 10.1093/eurheartj/ehad195, 37622666

[3] Li XH Yang XL Dong BB Liu Q. Predicting 28-day all-cause mortality in patients admitted to intensive care units with pre-existing chronic heart failure using the stress hyperglycemia ratio: a machine learning-driven retrospective cohort analysis. Cardiovasc Diabetol. (2025) 24:10. doi: 10.1186/s12933-025-02577-z 39780223 PMC11714879

[4] Seferovic PM Paulus WJ Rosano G Polovina M Petrie MC Jhund PS . Diabetic myocardial disorder. A clinical consensus statement of the heart failure Association of the esc and the esc working group on Myocardial & Pericardial Diseases. Eur J Heart Fail. (2024) 26:1893–903. doi: 10.1002/ejhf.3347 38896048

[5] Ni W Jiang R Xu D Zhu J Chen J Lin Y . Association between insulin resistance indices and outcomes in patients with heart failure with preserved ejection fraction. Cardiovasc Diabetol. (2025) 24:32. doi: 10.1186/s12933-025-02595-x, 39844150 PMC11755915

[6] Erqou S Adler AI Challa AA Fonarow GC Echouffo-Tcheugui JB. Insulin resistance and incident heart failure: a Meta-analysis. Eur J Heart Fail. (2022) 24:1139–41. doi: 10.1002/ejhf.2531, 35502564 PMC9262840

[7] Ho KL Karwi QG Connolly D Pherwani S Ketema EB Ussher JR . Metabolic, structural and biochemical changes in diabetes and the development of heart failure. Diabetologia. (2022) 65:411–23. doi: 10.1007/s00125-021-05637-7, 34994805

[8] Zhao X An X Yang C Sun W Ji H Lian F. The crucial role and mechanism of insulin resistance in metabolic disease. Front Endocrinol (Lausanne). (2023) 14:1149239. doi: 10.3389/fendo.2023.1149239, 37056675 PMC10086443

[9] Zhou Q Yang J Tang H Guo Z Dong W Wang Y . High triglyceride-glucose (Tyg) index is associated with poor prognosis of heart failure with preserved ejection fraction. Cardiovasc Diabetol. (2023) 22:263. doi: 10.1186/s12933-023-02001-4, 37775762 PMC10541699

[10] Kayan F Kömek H Kepenek F Yildirim MS. The role of the triglyceride–glucose index and other prognostic factors in predicting coronary slow flow. J Cardiovasc Dev Dis. (2026) 13:55. doi: 10.3390/jcdd13010055, 41590882 PMC12842290

[11] Kayan F Alakuş ÖF Elçiçek M Soner S Öztürk C Güleken G . The impact of body mass index and nutritional status on cardiac electrophysiological balance using Iceb and Icebc: a cross-sectional approach. J Cardiovas Develo. Dis. (2026) 13:109. doi: 10.3390/jcdd13030109, 41892698 PMC13027112

[12] Duan M Zhao X Li S Miao G Bai L Zhang Q . Metabolic score for insulin resistance (Mets-Ir) predicts all-cause and cardiovascular mortality in the general population: evidence from Nhanes 2001-2018. Cardiovasc Diabetol. (2024) 23:243. doi: 10.1186/s12933-024-02334-8, 38987779 PMC11238348

[13] Cho YR Ann SH Won KB Park GM Kim YG Yang DH . Association between insulin resistance, hyperglycemia, and coronary artery disease according to the presence of diabetes. Sci Rep. (2019) 9:6129. doi: 10.1038/s41598-019-42700-1, 31477741 PMC6718672

[14] Han S Wang C Tong F Li Y Li Z Sun Z . Triglyceride glucose index and its combination with the get with the guidelines-heart failure score in predicting the prognosis in patients with heart failure. Front Nutr. (2022) 9:950338. doi: 10.3389/fnut.2022.950338, 36159483 PMC9493032

[15] Nguyen HTT Ha TTT Tran HB Nguyen DV Pham HM Tran PM . Relationship between Bmi and prognosis of chronic heart failure outpatients in Vietnam: a single-center study. Front Nutr. (2023) 10:1251601. doi: 10.3389/fnut.2023.1251601, 38099185 PMC10720040

[16] Artola Arita V Beigrezaei S Franco OH. Risk factors for cardiovascular disease: the known unknown. Eur J Prev Cardiol. (2024) 31:e106–7. doi: 10.1093/eurjpc/zwad392, 38099566

[17] Yun YM. Apolipoprotein B, non-HDL cholesterol, and LDL cholesterol as markers for atherosclerotic cardiovascular disease risk assessment. Ann Lab Med. (2023) 43:221–2. doi: 10.3343/alm.2023.43.3.221, 36544332 PMC9791017

[18] Kang H Song J Cheng Y. Hdl regulates the risk of Cardiometabolic and inflammatory-related diseases: focusing on cholesterol efflux capacity. Int Immunopharmacol. (2024) 138:112622. doi: 10.1016/j.intimp.2024.112622, 38971111

[19] Mansoori A Nosrati M Dorchin M Mohammadyari F Derakhshan-Nezhad E Ferns G . A novel index for diagnosis of type 2 diabetes mellitus: cholesterol, high density lipoprotein, and glucose (Chg) index. J Diabetes Investig. (2025) 16:309–14. doi: 10.1111/jdi.14343, 39569998 PMC11786182

[20] Catak M Konuk SG Hepsen S. The cholesterol-Hdl-glucose (Chg) index and traditional adiposity markers in predicting diabetic retinopathy and nephropathy. J Diabetes Investig. (2025) 16:1487–94. doi: 10.1111/jdi.70086, 40434226 PMC12315246

[21] Xiao Q Huang W Zhuang Y Chen Y Peng G Li Y. Evaluating the role of Chg index in predicting stroke risk among adults with varying glucose regulation. Aging Clin Exp Res. (2025) 37:334. doi: 10.1007/s40520-025-03243-w, 41296126 PMC12657532

[22] Guo Z Xiong Z Zhang W Chen G Xie M Zhou Z . Association between cholesterol, high-density lipoprotein, and glucose index and risks of cardiovascular and all-cause mortality in patients with calcific aortic valve stenosis: the Aristotle cohort study. Cardiovasc Diabetol. (2025) 24:393. doi: 10.1186/s12933-025-02906-2, 41076530 PMC12514813

[23] Mo D Zhang P Zhang M Dai H Guan J. Cholesterol, high-density lipoprotein, and glucose index versus triglyceride-glucose index in predicting cardiovascular disease risk: a cohort study. Cardiovasc Diabetol. (2025) 24:116. doi: 10.1186/s12933-025-02675-y, 40065297 PMC11895360

[24] Suyoto PST Pamungkas NP de Vries JHM Feskens EJM. Associations between variability in between- and within-day dietary intake with adiposity and glucose homeostasis in adults: a systematic review. Adv Nutr. (2024) 15:100310. doi: 10.1016/j.advnut.2024.100310 39389469 PMC11566682

[25] Scaglione S Di Chiara T Daidone M Tuttolomondo A. Effects of the Mediterranean diet on the components of metabolic syndrome concerning the Cardiometabolic risk. Nutrients. (2025) 17:358. doi: 10.3390/nu17020358, 39861488 PMC11768522

[26] Pagidipati NJ Taub PR Ostfeld RJ Kirkpatrick CF. Dietary patterns to promote Cardiometabolic health. Nat Rev Cardiol. (2024) 22:38–46. doi: 10.1038/s41569-024-01061-7, 39020052

[27] Sun J Jiang X Li Z Shen Y. Impact of dietary patterns on the survival outcomes of patients with cardiovascular disease. Front Nutr. (2025) 12:1535174. doi: 10.3389/fnut.2025.1535174, 40808849 PMC12343251

[28] Johnson AEW Bulgarelli L Shen L Gayles A Shammout A Horng S . Mimic-iv, a freely accessible electronic health record dataset. Sci Data. (2023) 10:1. doi: 10.1038/s41597-022-01899-x, 36596836 PMC9810617

[29] Bates BA Akhabue E Nahass MM Mukherjee A Hiltner E Rock J . Validity of international classification of diseases (Icd)-10 diagnosis codes for identification of acute heart failure hospitalization and heart failure with reduced versus preserved ejection fraction in a National Medicare Sample. Circ Cardiovasc Qual Outcomes. (2023) 16:e009078. doi: 10.1161/CIRCOUTCOMES.122.009078, 36688301

[30] Che B Zhong C Zhang R Pu L Zhao T Zhang Y . Triglyceride-glucose index and triglyceride to high-density lipoprotein cholesterol ratio as potential cardiovascular disease risk factors: An analysis of Uk biobank data. Cardiovasc Diabetol. (2023) 22:34. doi: 10.1186/s12933-023-01762-2, 36797706 PMC9936712

[31] Dang K Wang X Hu J Zhang Y Cheng L Qi X . The association between triglyceride-glucose index and its combination with obesity indicators and cardiovascular disease: Nhanes 2003-2018. Cardiovasc Diabetol. (2024) 23:8. doi: 10.1186/s12933-023-02115-9, 38184598 PMC10771672

[32] Li H Zuo Y Qian F Chen S Tian X Wang P . Triglyceride-glucose index variability and incident cardiovascular disease: a prospective cohort study. Cardiovasc Diabetol. (2022) 21:105. doi: 10.1186/s12933-022-01541-5, 35689232 PMC9188105

[33] Lopez-Jaramillo P Gomez-Arbelaez D Martinez-Bello D Abat MEM Alhabib KF Avezum A . Association of the Triglyceride Glucose Index as a measure of insulin resistance with mortality and cardiovascular disease in populations from five continents (pure study): a prospective cohort study. Lancet Healthy Longev. (2023) 4:e23–33. doi: 10.1016/S2666-7568(22)00247-1, 36521498

[34] Zhang W Huo W Hu H Li T Yuan L Zhang J . Dose-response associations of triglyceride to high-density lipoprotein cholesterol ratio and triglyceride-glucose index with arterial stiffness risk. Lipids Health Dis. (2024) 23:115. doi: 10.1186/s12944-024-02095-z, 38643148 PMC11031917

[35] Dou J Guo C Wang Y Peng Z Wu R Li Q . Association between triglyceride glucose-body mass and one-year all-cause mortality of patients with heart failure: a retrospective study utilizing the Mimic-iv database. Cardiovasc Diabetol. (2023) 22:309. doi: 10.1186/s12933-023-02047-4, 37940979 PMC10634170

[36] Chen J-X Li Y Zhang Y-B Wang Y Zhou Y-F Geng T . Nonlinear relationship between high-density lipoprotein cholesterol and cardiovascular disease: An observational and Mendelian randomization analysis. Metabolism. (2024) 154:155817. doi: 10.1016/j.metabol.2024.155817, 38364900

[37] Stadler JT Marsche G. Dietary strategies to improve cardiovascular health: focus on increasing high-density lipoprotein functionality. Front Nutr. (2021) 8:761170. doi: 10.3389/fnut.2021.761170, 34881279 PMC8646038

[38] Charach G Argov O Nochomovitz H Rogowski O Charach L Grosskopf I. A longitudinal 20 years of follow up showed a decrease in the survival of heart failure patients who maintained low Ldl cholesterol levels. QJM. (2018) 111:319–25. doi: 10.1093/qjmed/hcy043, 29733423

[39] Simmonds SJ Cuijpers I Heymans S Jones EAV. Cellular and molecular differences between Hfpef and Hfref: a step ahead in an improved pathological understanding. Cells. (2020) 9:242. doi: 10.3390/cells9010242, 31963679 PMC7016826

[40] Thorp EB Filipp M. Contributions of inflammation to Cardiometabolic heart failure with preserved ejection fraction. Annu Rev Pathol. (2025) 20:143–67. doi: 10.1146/annurev-pathmechdis-111523-023405, 39357068 PMC12925995

[41] Ye M Feng S Xu Z He W Liu C Zhu W. Aging and metabolism in Hfpef: pathophysiology and therapeutic implications. Metabolism. (2025) 176:156460. doi: 10.1016/j.metabol.2025.156460, 41352525

[42] Meems LMG van Veldhuisen DJ Sattar N Lee MMY. Heart failure and obesity: novel insights leading to new treatment paradigms. Nat Rev Cardiol. (2025) 23:87–99. doi: 10.1038/s41569-025-01190-7, 40721643

[43] Sen P Sittig T Hamers J d'Ambrosio L Ornek I Zhang J . From kidney injury to cardiac dysfunction: the central role of oxidative stress in diabetes and CKD. Basic Res Cardiol. (2025) 121:93–112. doi: 10.1007/s00395-025-01153-6 41419687 PMC12804299

[44] Riccardi M Pabon MA Bhatt AS Savarese G Metra M Volterrani M . Heart failure with improved ejection fraction: definitions, epidemiology, and management. J Am Coll Cardiol. (2025) 85:2401–15. doi: 10.1016/j.jacc.2025.03.544, 40533130

[45] Duran EK Aday AW Cook NR Buring JE Ridker PM Pradhan AD. Triglyceride-rich lipoprotein cholesterol, small dense Ldl cholesterol, and incident cardiovascular disease. J Am Coll Cardiol. (2020) 75:2122–35. doi: 10.1016/j.jacc.2020.02.059, 32354380 PMC8064770

[46] Poznyak A Grechko AV Poggio P Myasoedova VA Alfieri V Orekhov AN. The diabetes mellitus-atherosclerosis connection: the role of lipid and glucose metabolism and chronic inflammation. Int J Mol Sci. (2020) 21:1835. doi: 10.3390/ijms21051835, 32155866 PMC7084712

[47] Sasko B Kelesidis T Kostin S Scharow L Mueller R Jaensch M . Reduced antioxidant high-density lipoprotein function in heart failure with preserved ejection fraction. Clin Res Cardiol. (2025) 115:232–40. doi: 10.1007/s00392-024-02583-3, 39812805 PMC12260025

[48] Yang M Shangguan Q Xie G Sheng G Yang J. Oxidative stress mediates the association between triglyceride-glucose index and risk of cardiovascular and all-cause mortality in metabolic syndrome: evidence from a prospective cohort study. Front Endocrinol (Lausanne). (2024) 15:1452896. doi: 10.3389/fendo.2024.1452896, 39229375 PMC11368748

[49] González-Domínguez Á Belmonte T Domínguez-Riscart J Ruiz-Ocaña P Muela-Zarzuela I Saez-Benito A . Altered insulin secretion dynamics relate to oxidative stress and Inflammasome activation in children with obesity and insulin resistance. J Transl Med. (2023) 21:559. doi: 10.1186/s12967-023-04337-7, 37599368 PMC10440893

[50] Chirinos JA Orlenko A Zhao L Basso MD Cvijic ME Li Z . Multiple plasma biomarkers for risk stratification in patients with heart failure and preserved ejection fraction. J Am Coll Cardiol. (2020) 75:1281–95. doi: 10.1016/j.jacc.2019.12.069, 32192654 PMC7147356

[51] D'Italia G Coenen DM Lemmens TP Brandts L Wielders SJH Nagy M . Immuno-Haemostatic dysregulation in heart failure with preserved ejection fraction. ESC Heart Fail. (2025) 12:3444–60. doi: 10.1002/ehf2.15361, 40641053 PMC12450788

[52] Mohammadi K Shafie D Ghomashi N Abdolizadeh A Sadeghpour M. Kinin-Kallikrein system: new perspectives in heart failure. Heart Fail Rev. (2024) 29:729–37. doi: 10.1007/s10741-024-10393-y, 38381277

[53] Jia H Ren F Liu H. Development of low glycemic index food products with wheat resistant starch: a review. Carbohydr Polym. (2025) 361:123637. doi: 10.1016/j.carbpol.2025.123637, 40368562

[54] Rabail R Altemimi AB Maerescu CM Socol CT Criste FL Khalid AR . Consumption of edible oil blended with flax, coconut, sunflower, and olive oil can significantly improve the negative health consequences of high-fat/high-cholesterol diet in Sprague Dawley rats. Front Nutr. (2024) 11:1469601. doi: 10.3389/fnut.2024.1469601, 39371945 PMC11452909

[55] Bugiardini R Rahaman T Manfrini O Maas A Bergami M Badimon L . Diet and sex inequities in ischemic heart disease mortality across Europe: findings from the global burden of disease study. Cardiovasc Res. (2025) 121:2432–46. doi: 10.1093/cvr/cvaf176, 41181878 PMC12687866

