# Supplementary material for: The cholesterol, high-density lipoprotein, and glucose index as a metabolic-nutritional biomarker for risk stratification in hospitalized heart failure patients
Source: Front Nutr. 2026 May 13;13:1805016. doi: 10.3389/fnut.2026.1805016 (PMC13213170; doi:10.3389/fnut.2026.1805016)
Supplement: Supplementary file 1 [file Table_1.docx]

**Supplementary materials**

Supplementary Table S1: **Unit conversion factors for laboratory parameters.**

Supplementary Table S2: **Baseline characteristics and outcomes of participants categorized by CHG index in training cohort.**

Supplementary Table S3: **Baseline characteristics and outcomes of participants categorized by CHG index in validation cohort.**

Supplementary Table S4: **AUC comparison of baseline and extended prediction models.**

Supplementary Table S5: **NRI and IDI for extended models versus the baseline model.**

Supplementary Figure S1. **Model performance of the CHG-extended model for predicting in-hospital mortality. (A)** Receiver operating characteristic curve of the CHG-extended model, with an AUC of 0.701 (95% CI 0.659–0.742); **(B)** Decision curve analysis of the baseline model and the CHG-extended model; **(C)** Calibration plot of the CHG-extended model. The dashed diagonal line indicates ideal calibration; the blue line indicates the bias-corrected curve, and the red line indicates the apparent curve. Abbreviation: CHG index, cholesterol, high-density lipoprotein, and glucose index; AUC, area under the curve; CI, confidence interval.

Table S1. **Unit conversion factors for laboratory parameters.**

| **Abbreviation** | **Original Unit** | **Target Unit** | **Conversion Factor** | **Conversion Formula** |
| --- | --- | --- | --- | --- |
| TC | mmol/L | mg/dL | 38.67 | mg/dL = mmol/L × 38.67 |
| HDL-C | mmol/L | mg/dL | 38.67 | mg/dL = mmol/L × 38.67 |
| LDL-C | mmol/L | mg/dL | 38.67 | mg/dL = mmol/L × 38.67 |
| TG | mmol/L | mg/dL | 88.57 | mg/dL = mmol/L × 88.57 |
| FBG | mmol/L | mg/dL | 18.02 | mg/dL = mmol/L × 18.02 |
| BUN | mmol/L | mg/dL | 2.801 | mg/dL = mmol/L × 2.801 |
| SCr | μmol/L | mg/dL | 0.01131 | mg/dL = μmol/L × 0.01131 |
| Hb | g/L | g/dL | 0.1 | g/dL=g/L × 0.1 |

Abbreviations: TC, total cholesterol; HDL-C, high-density lipoprotein cholesterol; LDL-C, low-density lipoprotein cholesterol; TG, triglyceride; FBG, fasting blood glucose; BUN, blood urea nitrogen; SCr, serum creatinine; Hb, hemoglobin

Table S2. **Baseline characteristics and outcomes of participants categorized by CHG index in training cohort.**

| **Variables** | **Total (n = 1,391)** | **Q1 (n = 348)** | **Q2 (n = 348)** | **Q3 (n = 348)** | **Q4 (n = 347)** | ***P* value** |
| --- | --- | --- | --- | --- | --- | --- |
| **Demographic** | | | | | | |
| Age, years | 73.23 (63.36, 82.93) | 78.41(67.18, 85.87) | 76.18 (65.60, 84.48) | 72.83 (63.78, 83.27) | 67.19 (58.27, 76.21) | <0.001 |
| Sex, n (%) | | | | | | <0.001 |
| Female | 620 (44.57) | 180 (51.72) | 175 (50.29) | 134 (38.51) | 131 (37.75) |  |
| Male | 771 (55.43) | 168 (48.28) | 173 (49.71) | 214 (61.49) | 216 (62.25) |  |
| Race, n (%) | | | | | | 0.665 |
| White | 839 (60.32) | 218 (62.64) | 219 (62.93) | 203 (58.33) | 199 (57.35) |  |
| Asian | 29 (2.08) | 7 (2.01) | 6 (1.72) | 10 (2.87) | 6 (1.73) |  |
| Black | 127 (9.13) | 32 (9.20) | 34 (9.77) | 30 (8.62) | 31 (8.93) |  |
| Others | 396 (28.47) | 91 (26.15) | 89 (25.57) | 105 (30.17) | 111 (31.99) |  |
| BMI, kg/m^2^, n (%) | | | | | | <0.001 |
| ≤30 | 406 (29.19) | 106 (30.46) | 100 (28.74) | 103 (29.60) | 97 (27.95) |  |
| >30 | 283 (20.35) | 40 (11.49) | 49 (14.08) | 86 (24.71) | 108 (31.12) |  |
| Missing | 702 (50.47) | 202 (58.05) | 199 (57.18) | 159 (45.69) | 142 (40.92) |  |
| **Comorbidities** | | | | | | |
| Hypertension, n (%) | 289 (20.78) | 66 (18.97) | 89 (25.57) | 67 (19.25) | 67 (19.31) | 0.089 |
| Diabetes, n (%) | 563 (40.47) | 87 (25.00) | 106 (30.46) | 151 (43.39) | 219 (63.11) | <0.001 |
| AMI, n (%) | 669 (48.09) | 117 (33.62) | 160 (45.98) | 178 (51.15) | 214 (61.67) | <0.001 |
| Renal Disease, n (%) | 476 (34.22) | 121 (34.77) | 108 (31.03) | 113 (32.47) | 134 (38.62) | 0.167 |
| Lung Disease, n (%) | 370 (26.60) | 96 (27.59) | 95 (27.30) | 99 (28.45) | 80 (23.05) | 0.376 |
| **Vital signs** | | | | | | |
| Heart Rate, bpm | 86.00 (73.00, 99.00) | 83.00 (71.00, 97.00) | 85.00 (72.00, 98.00) | 84.00 (72.00, 98.00) | 91.00 (77.25, 104.00) | <0.001 |
| SBP, mmHg | 129.00 (112.00, 148.00) | 130.00 (113.00, 148.00) | 129.00 (115.50, 145.00) | 129.00 (111.00, 147.00) | 130.00 (110.00, 150.00) | 0.898 |
| DBP, mmHg | 74.00 (63.00, 87.00) | 75.00 (62.50, 87.00) | 74.00 (63.00, 86.00) | 74.00 (63.00, 86.00) | 75.00 (63.25, 88.00) | 0.793 |
| SpO_2_, % | 97.00 (95.00, 99.00) | 97.00 (95.00, 99.50) | 97.00 (95.00, 100.00) | 97.00 (95.00, 99.00) | 97.00 (94.00, 99.00) | 0.285 |
| **Score, median (IQR)** | | | | | | |
| APS III | 41.00 (31.00, 52.00) | 39.00 (30.00, 50.25) | 38.00 (29.00, 48.00) | 40.00 (31.00, 52.25) | 44.00 (34.00, 56.00) | <0.001 |
| SOFA score | 4.00 (2.00, 7.00) | 4.00 (2.00, 6.00) | 3.50 (2.00, 6.00) | 4.00 (2.00, 7.00) | 4.00 (2.00, 8.00) | 0.009 |
| **Laboratory examination, median** | | | | | | |
| TC, mg/dL | 143.00 (114.00, 174.00) | 125.50 (102.75, 157.50) | 138.50 (115.00, 165.25) | 147.00 (119.75, 178.00) | 158.00 (124.00, 198.00) | <0.001 |
| TG, mg/dL | 98.00 (73.00, 136.00) | 78.00 (63.00, 95.00) | 94.00 (70.00, 120.00) | 107.00 (81.00, 148.50) | 131.00 (95.00, 208.00) | <0.001 |
| HDL-C, mg/dL | 43.00 (33.00, 53.00) | 52.00 (42.00, 64.00) | 46.00 (37.00, 55.25) | 41.00 (32.00, 50.00) | 35.00 (27.00, 44.00) | <0.001 |
| LDL-C, mg/dL | 73.00 (53.00, 101.00) | 57.00 (40.75, 79.25) | 71.00 (55.00, 93.00) | 81.50 (58.00, 108.00) | 93.00 (63.50, 126.50) | <0.001 |
| Glucose, mg/dL | 135.00 (108.00, 180.00) | 103.00 (89.00, 116.00) | 129.00 (109.00, 149.00) | 147.00 (122.00, 181.75) | 220.00 (167.00, 301.00) | <0.001 |
| HbA1c, % | 5.90 (5.50, 6.70) | 5.70 (5.30, 6.00) | 5.75 (5.50, 6.20) | 6.00 (5.50, 6.97) | 6.70 (5.90, 8.70) | <0.001 |
| TNT, μg/L | 0.28 (0.06, 1.75) | 0.13 (0.04, 0.48) | 0.20 (0.05, 1.60) | 0.43 (0.07, 2.16) | 0.55 (0.11, 2.56) | <0.001 |
| CK-MB, ng/mL | 7.00 (3.00, 32.00) | 5.00 (2.00, 12.50) | 6.00 (3.00, 21.25) | 10.00 (4.00, 43.00) | 10.00 (4.00, 67.00) | <0.001 |
| Albumin, g/dL | 3.50 (3.20, 3.80) | 3.50 (3.20, 3.80) | 3.50 (3.10, 3.80) | 3.60 (3.20, 3.90) | 3.40 (3.00, 3.80) | 0.313 |
| Creatinine, mg/dL | 1.10 (0.80, 1.60) | 1.10 (0.80, 1.50) | 1.10 (0.80, 1.50) | 1.10 (0.90, 1.60) | 1.20 (0.90, 1.80) | <0.001 |
| BUN, mg/dL | 22.00 (16.00, 34.00) | 21.00 (16.00, 33.00) | 21.00 (15.00, 33.00) | 22.00 (15.00, 34.00) | 24.00 (17.00, 35.50) | 0.089 |
| RBC, *10^12^/L | 3.99 ± 0.77 | 3.82 ± 0.74 | 3.93 ± 0.74 | 4.03 ± 0.75 | 4.18 ± 0.80 | <0.001 |
| Hb, g/dL | 11.84 ± 2.31 | 11.35 ± 2.18 | 11.74 ± 2.24 | 11.99 ± 2.32 | 12.28 ± 2.41 | <0.001 |
| Hematocrit, % | 36.25 ± 6.64 | 35.14 ± 6.21 | 35.97 ± 6.53 | 36.51 ± 6.45 | 37.39 ± 7.14 | <0.001 |
| WBC, *10^9^/L | 10.30 (7.70, 13.72) | 8.80 (7.00, 11.15) | 9.90 (7.50, 12.75) | 11.10 (8.00, 14.50) | 12.40 (9.43, 16.48) | <0.001 |
| Platelet, *10^9^/L | 214.50 (168.00, 272.75) | 194.00 (152.00, 247.50) | 214.50 (168.00, 266.25) | 217.50 (168.00, 277.75) | 234.00 (182.00, 292.50) | <0.001 |
| CHG index | 5.44 (5.09, 5.84) | 4.87 (4.70, 4.99) | 5.27 (5.19, 5.35) | 5.63 (5.53, 5.71) | 6.17 (6.00, 6.48) | <0.001 |
| **Events** | | | | | | |
| In-hospital mortality, n, (%) | | | | | | 0.006 |
| 0 | 1209 (86.92) | 317 (91.09) | 302 (86.78) | 305 (87.64) | 285 (82.13) |  |
| 1 | 182 (13.08) | 31 (8.91) | 46 (13.22) | 43 (12.36) | 62 (17.87) |  |
| Hospital length of stay, days | 7.92 (4.61, 13.88) | 7.76 (4.69, 14.46) | 7.77 (4.75, 13.10) | 7.36 (3.92, 13.61) | 8.90 (5.13, 14.58) | 0.062 |

Abbreviations: CHG index, cholesterol, high-density lipoprotein, and glucose index; BMI, body mass index; AMI, acute myocardial infarction; SBP, systolic blood pressure; DBP, diastolic blood pressure; SpO_2_, peripheral oxygen saturation; APS III, Acute Physiology Score III; SOFA, Sequential Organ Failure Assessment; TC, total cholesterol; TG, triglyceride; HDL-C, high-density lipoprotein cholesterol; LDL-C, low-density lipoprotein cholesterol; HbA1c, glycated hemoglobin; TNT, troponin T; CK-MB, creatine kinase MB; BUN, blood urea nitrogen; RBC, red blood cell count; Hb, hemoglobin; WBC, white blood cell count

Table S3. **Baseline characteristics and outcomes of participants categorized by CHG index in validation cohort.**

| **Variables** | **Total (n=296)** | **Q1 (n = 74)** | **Q2 (n = 74)** | **Q3 (n = 74)** | **Q4 (n = 74)** | ***P* value** |
| --- | --- | --- | --- | --- | --- | --- |
| **Demographic** | | | | | | |
| Age, years | 75.50 (64.75, 82.00) | 79.00 (72.00,84.00) | 77.50 (67.25,83.00) | 73.00 (60.25,79.00) | 72.50 (57.00,79.00) | 0.001 |
| Sex, n (%) |  |  |  |  |  | 0.604 |
| Female | 117 (39.53) | 29 (39.19) | 34 (45.95) | 27 (36.49) | 27 (36.49) |  |
| Male | 179 (60.47) | 45 (60.81) | 40 (54.05) | 47 (63.51) | 47 (63.51) |  |
| BMI, kg/m^2^, n (%) |  |  |  |  |  | 0.193 |
| ≤30 | 185 (62.50) | 54 (72.97) | 45 (60.81) | 45 (60.81) | 41 (55.41) |  |
| >30 | 25 (8.45) | 3 (4.05) | 6 (8.11) | 10 (13.51) | 6 (8.11) |  |
| Missing | 86 (29.05) | 17 (22.97) | 23 (31.08) | 19 (25.68) | 27 (36.49) |  |
| **Comorbidities** | | | | | | |
| Hypertension, n (%) | 222 (75.00) | 55 (24.77) | 54 (24.32) | 57 (25.68) | 56 (25.23) | 0.948 |
| Diabetes, n (%) | 140 (47.30) | 19 (13.57) | 26 (18.57) | 38 (27.14) | 57 (40.71) | <0.001 |
| AMI, n (%) | 36 (12.16) | 11 (30.56) | 4 (11.11) | 9 (25.00) | 12 (33.33) | 0.187 |
| Renal Disease, n (%) | 123 (41.55) | 28 (22.76) | 30 (24.39) | 26 (21.14) | 39 (31.71) | 0.139 |
| Lung Disease, n (%) | 17 (5.74) | 4 (23.53) | 7 (41.18) | 5 (29.41) | 1 (5.88) | 0.181 |
| **Vital signs** | | | | | | |
| Heart Rate, bpm | 93.00 (82.00, 107.00) | 90.00 (78.00,104.00) | 91.50 (78.50,104.75) | 99.00 (85.00,113.00) | 94.00 (84.00,106.25) | 0.099 |
| SBP, mmHg | 139.57 ± 27.04 | 138.04 ± 23.64 | 141.63 ± 26.55 | 141.67 ± 28.47 | 136.83 ± 29.37 | 0.612 |
| DBP, mmHg | 79.00 (67.00, 93.00) | 75.50 (67.00,87.50) | 80.00 (71.00,94.00) | 81.00 (72.00,95.00) | 74.00 (64.50,89.50) | 0.048 |
| SpO_2_, % | 97.00 (95.00, 99.00) | 98.00 (95.00,99.00) | 97.00 (93.00,99.00) | 97.00 (95.00,99.00) | 97.00 (94.00,99.00) | 0.262 |
| **Laboratory examination, median (IQR)** | | | | | | |
| TC, mg/dL | 141.15 (114.85, 168.41) | 125.29 (107.31,151.88) | 137.09 (107.41,155.26) | 141.34 (118.33,176.92) | 156.81 (138.44,207.75) | <0.001 |
| TG, mg/dL | 91.23 (69.97, 125.11) | 73.07 (62.00,95.43) | 84.14 (70.19,107.17) | 95.66 (72.41,126.43) | 124.00 (95.88,167.40) | <0.001 |
| HDL-C, mg/dL | 38.28 (30.55, 48.05) | 44.86 (37.41,55.97) | 40.60 (32.10,49.11) | 36.35 (30.55,43.70) | 32.10 (26.88,38.96) | <0.001 |
| LDL-C, mg/dL | 86.81 (65.55, 107.12) | 72.12 (53.75,86.43) | 79.27 (61.20,97.26) | 88.75 (70.48,114.27) | 106.73 (89.52,130.41) | <0.001 |
| Glucose, mg/dL | 138.78 (107.33, 198.00) | 98.73 (86.35,114.98) | 128.61 (112.91,150.79) | 157.95 (120.78,198.00) | 226.35 (174.47,261.54) | <0.001 |
| HbA1c, % | 6.10 (5.70, 6.95) | 5.80 (5.50,6.10) | 6.00 (5.60,6.50) | 6.30 (5.80,7.45) | 7.05 (6.10,8.40) | <0.001 |
| Albumin, g/dL | 3.52 ± 0.47 | 3.60 ± 0.41 | 3.53 ± 0.49 | 3.48 ± 0.45 | 3.49 ± 0.53 | 0.350 |
| Creatinine, mg/dL | 1.16 (0.85, 1.87) | 1.07 (0.84,1.49) | 1.14 (0.91,1.57) | 1.07 (0.76,1.76) | 1.49 (1.01,2.53) | 0.025 |
| BUN, mg/dL | 26.60 (18.13, 40.88) | 26.04 (19.11,35.14) | 28.28 (19.88,38.85) | 23.10 (16.73,35.56) | 33.46 (17.71,56.63) | 0.068 |
| RBC, 10^12^/L | 3.91 (3.28, 4.53) | 3.90 (3.38,4.51) | 3.82 (2.94,4.52) | 4.07 (3.44,4.67) | 3.90 (3.35,4.49) | 0.411 |
| Hemoglobin, g/dL | 11.70 (9.70, 13.53) | 12.15 (10.22,13.30) | 11.50 (8.83,13.20) | 11.70 (10.05,14.40) | 11.25 (9.57,13.55) | 0.438 |
| Hematocrit | 35.70 (30.25, 41.02) | 36.75 (31.10,40.27) | 35.70 (27.47,40.98) | 36.05 (31.38,43.88) | 34.30 (29.40,40.38) | 0.673 |
| WBC, 10^9^/L | 8.39 (5.90, 11.37) | 7.42 (5.41,9.45) | 8.13 (5.74,10.88) | 8.57 (6.19,11.25) | 9.52 (6.71,13.43) | 0.006 |
| Platelet, 10^9^/L | 185.00 (137.00, 238.25) | 162.50 (129.25,206.00) | 160.00 (130.50,211.50) | 188.00 (147.00,260.50) | 217.00 (156.50,282.25) | <0.001 |
| CHG index | 5.52 (5.25, 5.95) | 5.00 (4.83,5.12) | 5.39 (5.33,5.45) | 5.69 (5.60,5.81) | 6.27 (6.08,6.56) | <0.001 |
| **Events** | | | | | | |
| Hospital mortality, n, (%) | | | | | | 0.010 |
| 0 | 273 (92.23) | 71 (95.95) | 72 (97.30) | 68 (91.89) | 62 (83.78) |  |
| 1 | 23 (7.77) | 3 (4.05) | 2 (2.70) | 6 (8.11) | 12 (16.22) |  |
| Hospital length of stay, days | 10.00 (8.00, 13.00) | 9.00 (7.00,13.00) | 10.00 (8.00,13.00) | 9.50 (8.00,12.75) | 10.00 (8.00,14.00) | 0.659 |

Abbreviations: CHG index, cholesterol, high-density lipoprotein, and glucose index; BMI, body mass index; AMI, acute myocardial infarction; SBP, systolic blood pressure; DBP, diastolic blood pressure; SpO_2_, peripheral oxygen saturation; TC, total cholesterol; TG, triglyceride; HDL-C, high-density lipoprotein cholesterol; LDL-C, low-density lipoprotein cholesterol; HbA1c, glycated hemoglobin; BUN, blood urea nitrogen; RBC, red blood cell count; Hb, hemoglobin; WBC, white blood cell count

Table S4. **AUC comparison of baseline and extended prediction models.**

| **Model** | **AUC** | **95% CI** | **ΔAUC vs baseline** |
| --- | --- | --- | --- |
| Baseline model | 0.683 | 0.641–0.725 | – |
| Baseline model + TyG index | 0.697 | 0.657–0.738 | 0.014 |
| Baseline model + CHG index | 0.701 | 0.659–0.742 | 0.018 |

Abbreviations: AUC, area under the curve; CI, confidence interval; TyG index, triglyceride-glucose index; CHG index, cholesterol, high-density lipoprotein, and glucose index

Table S5. **NRI and IDI for extended models versus the baseline model.**

| **Extended model compared with baseline** | **Categorical NRI (95% CI), *P* value** | **Continuous NRI (95% CI), *P* value** | **IDI (95% CI), *P* value** |
| --- | --- | --- | --- |
| Baseline model + TyG index | 0.018 (-0.013–0.049, *P* = 0.256) | 0.228 (0.068–0.389, *P* = 0.005) | 0.010 (0.004–0.017, *P* = 0.003) |
| Baseline model + CHG index | 0.033 (-0.005–0.072, *P* = 0.091) | 0.171 (0.011–0.331, *P* = 0.036) | 0.019 (0.010–0.029, *P* < 0.001) |

Abbreviations: NRI, net reclassification improvement; IDI, integrated discrimination improvement; CI, confidence interval; TyG index, triglyceride-glucose index; CHG index, cholesterol, high-density lipoprotein, and glucose index





Figure S1. **Model performance of the CHG-extended model for predicting in-hospital mortality. (A)** Receiver operating characteristic curve of the CHG-extended model, with an AUC of 0.701 (95% CI 0.659–0.742); **(B)** Decision curve analysis of the baseline model and the CHG-extended model; **(C)** Calibration plot of the CHG-extended model. The dashed diagonal line indicates ideal calibration; the blue line indicates the bias-corrected curve, and the red line indicates the apparent curve. Abbreviation: CHG index, cholesterol, high-density lipoprotein, and glucose index; AUC, area under the curve; CI, confidence interval.
